# A Case Report of Presumptive Primary Lymphedema Localized to the Face of a Dog

**DOI:** 10.3390/vetsci10070409

**Published:** 2023-06-22

**Authors:** Nina Poláková, Kristina Anna Lederer, Barbara Richter, Lucia Panáková

**Affiliations:** 1 Clinical Department for Small Animals and Horses, University of Veterinary Medicine Vienna, 1210 Vienna, Austria; lucia.panakova@vetmeduni.ac.at; 2 Clinical Unit of Diagnostic Imaging, University of Veterinary Medicine Vienna, 1210 Vienna, Austria; kristina.lederer@vetmeduni.ac.at; 3 Institute of Pathology, University of Veterinary Medicine Vienna, 1210 Vienna, Austria; barbara.richter@vetmeduni.ac.at

**Keywords:** lymphedema, computed tomography, indirect CT-lymphography, dog

## Abstract

**Simple Summary:**

Canine primary lymphedema is a rare condition, mostly observed in young dogs as an edema of the hind leg(s). There are only a few reports of this condition, but the patients generally have considerably impaired quality of life, and their prognosis is usually guarded. This case report describes clinical, radiographic, and histologic findings of a one-year-old German Shorthair Pointer with a unique presentation of a presumed primary lymphedema localized to the muzzle with a mild course of disease.

**Abstract:**

Primary lymphedema (PLE) is an uncommon diagnosis in veterinary medicine, with most of the previously described cases showing lower limb edema associated with a guarded long-term prognosis. To the authors’ knowledge, this case report describes the first case of lymphedema localized unilaterally to the facial region of one-year-old German Shorthair Pointer, in which indirect CT-lymphography, combined with histopathologic examination of the skin, resulted in a tentative diagnosis of PLE.

## 1. Introduction

Lymphedema is defined as a condition in which capillary filtration exceeds fluid and protein reabsorption by the venous and lymphatic systems, resulting in lymphatic congestion and edema [1,2,3]. Primary lymphedema occurs in patients with a congenital abnormality or dysfunction of the lymphatic system [2].

Few publications describe PLE in dogs, cats, cattle, and pigs, and most of them use clinical, necropsy, or radiographic findings, with or without the use of contrast medium to establish the diagnosis [1,4,5,6,7,8,9,10,11,12,13,14]. In dogs with PLE, the disease begins in the first days to months of life, and the most common clinical presentation is unilateral or bilateral pitting edema of the hind limbs [1,6]. Other parts of the body may also be affected, but this has been described only in combination with lymphedema of distal limb(s) [1,5,6,8]. Aplastic lymph nodes have been suspected in many dogs with PLE, whereas a recent study used computed tomography (CT) to demonstrate aplasia of multiple lymphoid structures [1,5]. Histopathologic changes in lymphedematous skin have been described in some of these studies, but no report has discussed histologic findings to any great extent.

Conservative and surgical therapy for dogs with PLE has been described previously [4,5,15,16], but the prognosis in general has been guarded to poor, in part because of complicating secondary infections. To the authors’ knowledge, this case report describes the first case of lymphedema localized unilaterally to the facial region of a dog in which indirect CT-lymphography combined with histopathologic examination of the skin resulted in a tentative diagnosis of PLE.

## 2. Case Report

**Figure 1:**
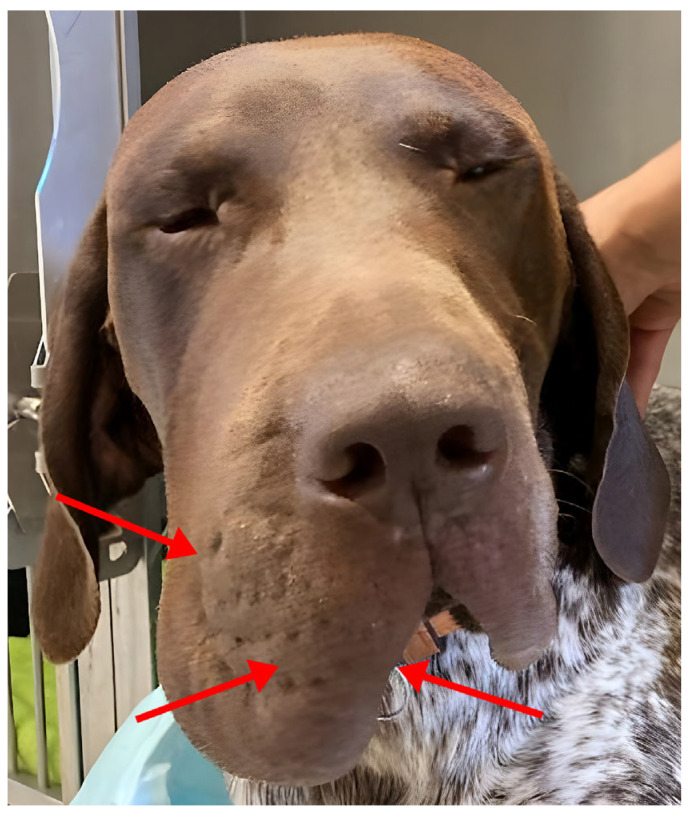
A one-year-old male German Shorthair Pointer. Note the moderate diffuse edema of the right muzzle. The arrows indicate the area where the biopsy samples were taken.

A one-year-old male German Shorthair Pointer was presented to the Dermatology Service of the University of Veterinary Medicine, Vienna, because of a unilateral, painless swelling of the muzzle that had persisted for three months (Figure 1). According to the owner, the condition occurred suddenly, although subsequent discussions with owners indicated the swelling occurred within a few days to weeks, with no known prior injury to the area. On inquiry, no other relatives of the dog showed similar clinical signs.

The patient had not received any medications or vaccinations prior to or at the time of the occurrence of the lesion, but had received an oral fluralaner (Bravecto^®^, MSD Animal Health, Rahway, NJ, USA) two weeks prior to presentation to the author’s clinic. This treatment had no effect on clinical signs.

Physical examination revealed moderate pitting edema, extending over a large area of the right muzzle and cheek. Examination of the oral cavity and auditory canals revealed no abnormalities. Apart from the described findings, the physical examination was unremarkable.

Differential diagnoses included primary lymphedema (congenital), versus secondary lymphedema (due to a trauma, dirofilariosis, or neoplasia), as well as other less likely conditions, such as an abscessation, neoplasm, hematoma, or cyst.

A routine blood count and biochemistry profile were performed, and, except for a mild eosinophilia, the findings were unremarkable. Fine-needle aspiration of the swelling revealed only a few drops of clear fluid, and cytologic slides stained with modified Wright-Giemsa stain (Haema-Schnellfärbung LT-SYS^®^ (Diff Quick), Labor + Technik Eberhard Lehmann GmbH, Berlin, Germany) contained only a few erythrocytes, but no other cells or microorganisms.

The CT of the head, neck, and thorax revealed marked diffuse subcutaneous low-attenuation swelling of the right upper lip and cheek, extending caudally to the laryngeal region, where subcutaneous fat stranding was identified. The main finding was a small right mandibular lymph node (3.5 mm in height) compared to the left side (10 mm in height). The size of all other lymph nodes was considered appropriate for the patient’s age and symmetry.

**Figure 2:**
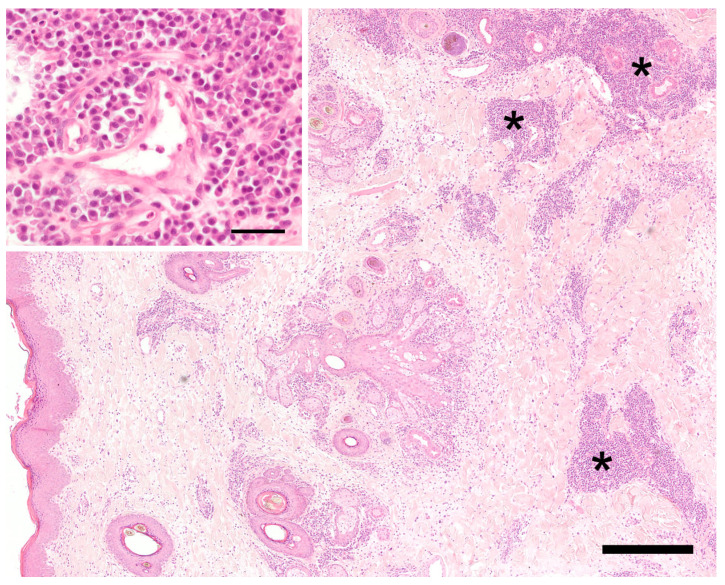
Histology of the haired skin of the lip. The superficial and mid-dermis on the left show a moderate diffuse edema and dilated vascular and lymphatic vessels. On the right side, in the deep dermis and subcutis, there are perivascular and periadnexal, mainly plasmacellular inflammatory infiltrates (asterisks). Hematoxylin-eosin. Bar = 400 µm. Inset: perivascular plasmacells. Hematoxylin-eosin. Bar = 40 µm.

**Figure 3:**
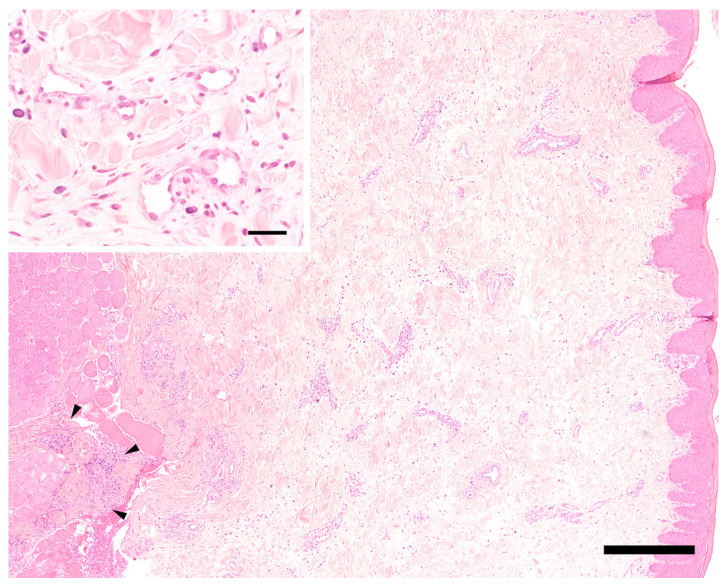
Histology of the mucosa of the lip. Moderate diffuse edema is visible in the center and on the right side corresponding to submucosa and mucosa. Only few mixed perivascular inflammatory infiltrates are present, extending focally into the striated muscle on the left side (arrowheads). Hematoxylin-eosin. Bar = 400 µm. Inset: small caliber venous and/or lymphatic vessels are lined by plump endothelial cells. The vessel walls are otherwise unchanged. Note extensive clear spaces between tissue structures (extracellular edema). Hematoxylin-eosin. Bar = 40 µm.

Three eight milimeter punch biopsies were taken from the affected skin and mucosa, and additional bacterial and fungal tissue cultures were collected in a sterile manner from the deep portion of the swelling. Histopathology showed diffuse changes in the dermis/mucosa and subcutis/submucosa extending partially into the perimysium of the striated muscle (Figure 2 and Figure 3). These changes consisted of moderate diffuse edema, mild diffuse fibroplasia, and inflammatory infiltrates that were present in various amounts. These inflammatory infiltrates were mostly perivascular and periadnexal. In one skin sample, they were present multifocally to confluently in the deep dermis and subcutis. They consisted mainly of plasma cells, moderate numbers of eosinophils, and mast cells, but they only consisted of fewer neutrophils, lymphocytes, and rare histiocytes. Histologically, it was hardly possible to discern lymphatic from venous vessels. The number of lymphatic vessels appeared to be normal, but it could not be assessed objectively, since there was no control tissue, e.g., from the contralateral side, available. The endothelium of blood and lymph vessels was plump and activated, and the vessels were moderately dilated. The walls of the presumed lymphatic vessels did not show obvious structural changes. The interfascicular connective tissue of the striated muscle was multifocally infiltrated by similar cells as the dermis, but to a lesser degree. Additionally, the edema was very mild in the deeper tissues, and it was more apparent in the dermis. Few small pustules containing neutrophils and eosinophils were present in the focally moderately hyperplastic epidermis of the haired skin. Overall, a chronic–active, deep, perivascular to nodular plasmacytic and eosinophilic cheilitis with diffuse edema was diagnosed.

Aerobic and anaerobic bacterial and fungal cultures were negative. Fine-needle aspirates from the mandibular and retropharyngeal lymph nodes on the contralateral side of the swelling showed no abnormalities.

A new list of differential diagnoses was established, considering any pathology of vessels—lymphatic and/or blood vessels—in the affected area. These included vessel obstruction (sterile—due to thrombi secondary to traumatic event; or, they may be infectious, e.g., filarial infection), dysfunction, or aplasia of lymphatic structures.

An ultrasound examination, including Doppler ultrasonography for evaluation of the vasculature, did not reveal any vascular thrombi. A real-time PCR of a whole blood sample for microfilaria organisms (Laboklin GMBH & CO.KG Bad Kissingen, Germany) was negative. This method is able to detect *D. repens*, *D. immitis, A. reconditum,* and *A. dracunculoides* infections.

Finally, an indirect CT lymphography was performed under general anesthesia with iodinated contrast medium. A total amount of 10 mL of contrast media (Ioversol, Optiray 300 mg I/mL, Guerbet) was injected subcutaneously into the rostral aspect of the lip on both sides over a period of two minutes, followed by a massage of that area of the same duration. Thirty minutes after the application of contrast agent, a total of seven post-injection scans were performed.

**Figure 4:**
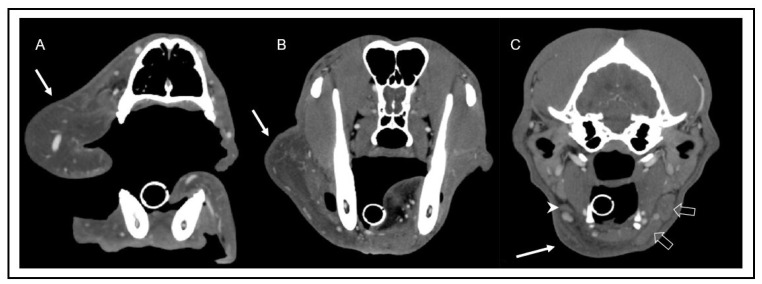
Transverse computed tomography images after intravenous contrast media injection in soft tissue window at the level of the mid nasal cavity (**A**), the frontal sinus and mandibular ramus (**B**), and the tympanic bulla (**C**). Note the marked low attenuation swelling consistent with subcutaneous edema (arrow) of the right lip (**A**) and neck (**B**), extending caudoventrally to the region of the mandibular lymph nodes (**C**). In image (**C**), the hypoplasia of the right mandibular lymph nodes (arrowhead) can be appreciated in comparison to the left mandibular lymph nodes (open arrows).

**Figure 5:**
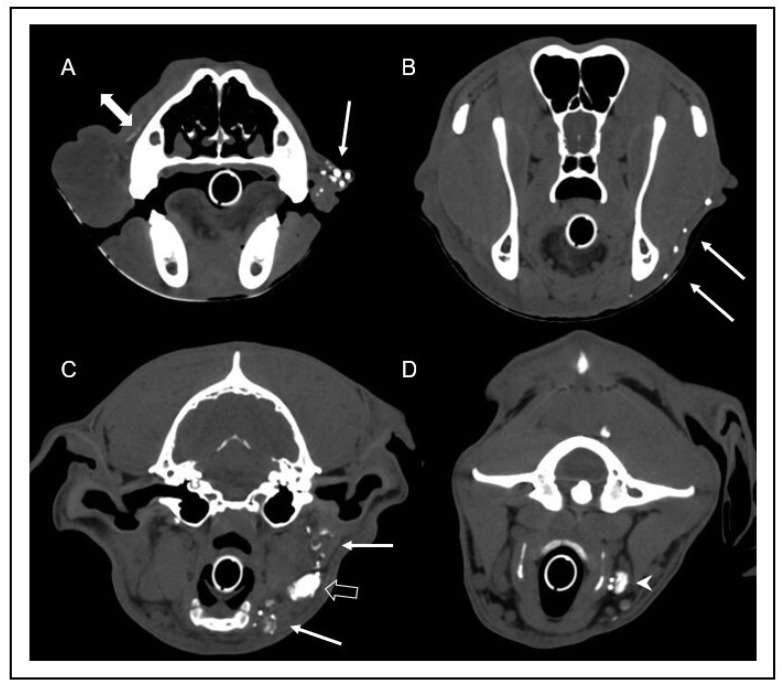
Transverse computed tomographic images after indirect lymphography with subcutaneous injection of contrast medium into the rostral aspect of the bilateral lips one minute after injection. Images were obtained at the level of the rostral nasal cavity (**A**), frontal sinus (**B**), tympanic bulla (**C**), and atlas (**D**). Multiple well enhanced lymphatic vessels (arrows) can be identified in the left lip (**A**), continuing caudoventrally (**B**), and draining into the left mandibular lymph nodes (open arrow in image (**C**)) and the left medial retropharyngeal lymph node (arrowhead in image (**D**)). Note the scant volume of contrast media extending along the facial planes (double-headed arrow in image (**A**)). On the right side, lymphatic vessels could not be identified, and there was no contrast enhancement of the right mandibular and medial retropharyngeal lymph nodes.

Immediately after injection, multiple lymphatic vessels were visualized on the left side, draining to the ipsilateral mandibular and medial retropharyngeal lymph nodes. On the right side, a scant volume of contrast media was identified, extending caudally from the injection site along the facial planes. No lymphatic vessels or lymph node enhancement could be appreciated on the right side in any scan (Figure 4 and Figure 5). In summary, there was absent lymphatic drainage of the right upper lip/cheek, and the small right mandibular lymph nodes were considered to be hypoplastic. In light of these findings, the most likely diagnosis was a primary lymphedema due to absent or malfunctioning lymphatic vessels.

**Figure 6:**
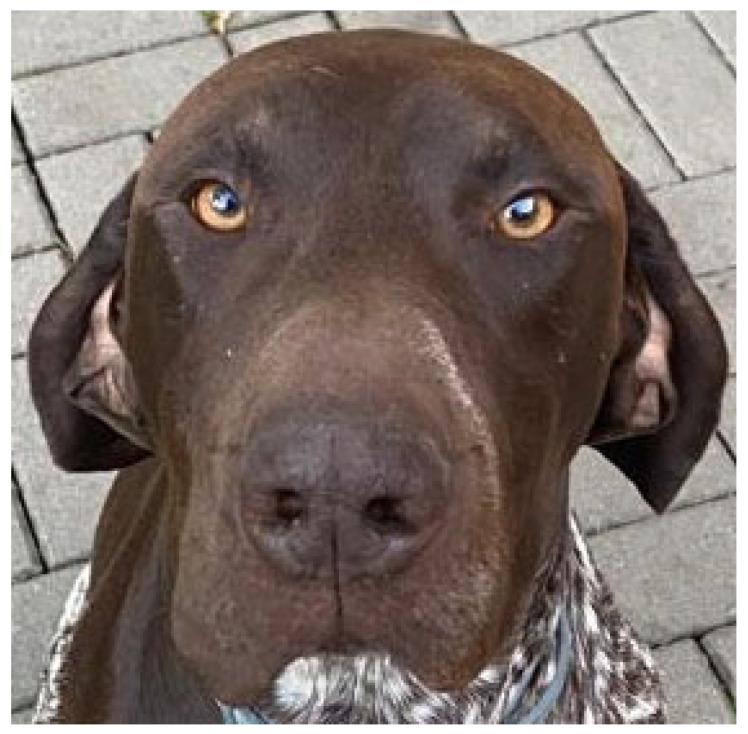
The same dog as in Figure 1, one year after the initial presentation.

While waiting for the laboratory results, therapy with oral doxycycline 5 mg/kg twice daily was initiated, but it did not result in any improvement within one week. After obtaining negative tissue culture results and histopathology report describing severe inflammation of the deep dermis and subcutis with fibroplasia, oral prednisolone in the dose of 1 mg/kg SID was administered. This resulted in a partial decrease in swelling of approximately 60% within the first week. After no further improvement, prednisolone was discontinued. A veterinary physical therapist began lymphatic drainage massage therapy, which was continued by the owner. More than a year after initial presentation, the owner reports a good quality of life for the dog, with ongoing lymphatic drainage therapy, but without medication, resulting in only mild to moderate edema of the lip (Figure 6).

## 3. Discussion

In human medicine, Milory’s disease and Meige’s disease are two forms of congenital lymphedema in pediatric patients that differ in age of onset and genetic background [2]. Autosomal dominant inheritance of mutations in the vascular endothelial growth factor receptor (VEGFR) of lymphatic vessels and in the FOXC2 gene involved in adipocyte metabolism is described in Milory disease and Meige disease, respectively [2,3,17,18,19,20]. In contrast to the abundant information on these human conditions, there is only a handful of reports of PLE in animals [1,4,5,6,7,8,9,10,11,12,13,14]. None of these studies performed a genome analysis of animals with PLE, but one study found an autosomal dominant mode of inheritance in a colony of affected mix-breed dogs [6]. In our case, no related dogs were affected, and as there is no genetic test available commercially, the diagnosis of PLE in this dog cannot be confirmed.

In human pediatric PLE, the age of onset is in the first two years of life for Milory’s disease and around puberty for Meige’s disease [2]. Most reported cases of PLE in dogs manifest at birth or within the first few weeks of life [1]. Only occasionally, the age of onset was greater than six months [1]. The lymphedema in the patient described here developed in the seventh month of life. It is possible, although unlikely, that mild swelling of the muzzle had been present for some time and went unnoticed by the owner. As will be discussed later, a concurrent insect bite in this area could possibly exacerbate this condition and lead to an acute presentation in the owner’s eye.

The reported distribution of lymphedema in humans and dogs affected by PLE is largely identical with pitting edema, occurring most commonly in the distal legs and hind limbs, respectively [1,2,6]. In dogs, both hind limbs are usually affected, but unilateral presentations have also been observed [1]. In severe cases, involvement of all limbs, as well as the head, ventrum, and tail, has been reported [1,5]. However, sites other than the extremities have only been affected in association with lymphedema of the distal limb(s), and this never presents alone. In contrast to these previous findings, our case showed localized lymphedema of the muzzle and facial area without affecting other parts of the body. Because of this unusual appearance, the authors initially suspected secondary lymphedema and attempted to exclude its potential primary cause, such as neoplasia, thrombosis, and especially filariasis.

Secondary lymphedema is a common condition in humans, with most cases in developed countries occurring due to malignancy or its therapy [2]. Histopathologic sections of our patient’s lip showed no evidence of autonomous growth. Furthermore, clinical symptoms were in partial remission more than one year after initial presentation. In combination, these findings make neoplasia highly unlikely, as the primary cause of lymphedema in this patient. Additionally, no surgical procedures have been carried out in the affected area.

Worldwide, the most common cause of secondary lymphedema in humans is filariasis, with more than 51 million individuals affected in 2018 [2,21]. The most commonly implicated nematodes in human lymphatic filariasis are *Wuchereria bancrofti*, *Brugia malayi*, and *Brugia timori* [22]. Lymphatic filariasis is known to be a tropical disease, mostly prevalent in America, Southeast Asia, and Africa [21].

To the authors’ knowledge, no case of filarial lymphedema in dogs has been reported. The patient described in this case report lived prior to presentation in Slovakia, which is an endemic region for *Dirofilaria immitis,* with sporadic occurrence of *Dirofilaria repens* [23,24]. Microfilaria PCR was negative, no filaria treatment was given previously, and no filarial organisms could be found on histology, even when recuts of the samples were examined.

Other infectious agents, particularly tuberculosis and chlamydial infections, have been reported in the pathogenesis of lymphedema in humans [25]. In our patient, there are no signs suggestive of such etiology.

Chronic Venous Insufficiency (CVI) is a diseases of lower limbs in humans [26]. It can lead to lymphedema, but also to ulcerations and thrombosis [25,26]. The exact etiology is unknown, but a combination of genetic predisposition and lifestyle factors, such as prolonged standing, obesity, pregnancy, and diet, are suspected [26]. Lymphatic vessels show structural changes in histopathologic sections of skin of CVI patients, consisting of the collapse of the lumen, loss of open intercellular junctions, and derangement of anchoring filaments [27]. None of the changes described above apply to our patient, and, therefore, a similar etiology is highly unlikely.

It is recognized that chronic inflammatory skin diseases, such as rheumatoid arthritis, psoriasis, or sarcoidosis, can cause lymphedema in humans [25]. Our patient did not exhibit any other skin changes or symptoms at the time of presentation or during the follow-up period of more than a year, making this an unlikely cause of lymphedema.

Another common cause of secondary lymphedema in humans is post-traumatic lymphedema [28]. The majority of reported cases describe a fracture as initiating event, but also burns, penetrating, and blunt injuries, resulting in the disruption of the cisterna chyli, thoracic duct, or other regions of increased density of lymphatic vessels, e.g., extremities are reported. The onset of lymphedema may be delayed for up to four months after the traumatic event [29]. In our case, there was neither prior history of trauma, nor clinical findings, suggesting a previous injury. These data, together with the results of indirect CT lymphography of the patient and the fact that minor trauma rarely leads to chronic lymphedema, make this mechanism of development of lymphedema in this dog highly unlikely [28].

Similarly to human patients with Milory’s disease, previously described cases of canine PLE have shown hypoplasia and/or aplasia of lymphatic tissues. In most studies in dogs, these findings were based on palpation, radiographic lymphangiography, or necropsy [6]. One recent study, however, used computed tomography to visualize multifocal hypoplasia and aplasia of lymphatic tissues [5]. The patient described in the current report was considered to have hypoplastic unilateral mandibular lymph nodes.

Histopathologic findings in cases of PLE have been previously described in human medicine as a combination of dilated dermal lymphatic capillaries with partly dilated interendothelial spaces, intra- and pericapillary edema, and variable dermal inflammatory infiltrate with histiocytes, plasma cells, and lymphocytes [30]. Hyperkeratosis and fibrosis can also be present and are more pronounced in a chronic stage of the disease [30,31,32,33]. The only report from veterinary medicine is a recent publication of a Great Dane puppy with generalized PLE. Here, histopathologic changes of the skin are described as myxedema with mild lymphocytic infiltration [5]. Our findings of diffuse edema of skin and subcutis together with activated endothelium, and moderate amounts of inflammatory infiltrates are in accordance with reported lymphedema [32]. Thin-walled vessels without intraluminal erythrocytes, interpreted to be lymph vessels, appeared to be moderately dilated, but structurally normal. Distinct ectasia, contraction, or sclerosis of the lymphatic vessel wall, as described by Barone et al., were not visible [34]. However, an unequivocal identification of the lymph vessels using immunohistochemistry was not attempted. Thus, the histological picture supports a diffuse lymphatic edema and could be in accordance with a localized hypoplasia of lymph vessels in the edematous muzzle, as seen in indirect lymphography.

Neither in human, nor in veterinary medicine, has a mixed infiltration with eosinophilic granulocytes and mast cells been described as a feature of lymphedema. One possible explanation for the presence of these cells in the dermis of our patient could be a previous insect bite on the affected area. Even though no such incident was observed by the owner, it could explain an acute onset of the swelling. The mild eosinophilia was no longer present on reexamination one month later, so no further investigations were performed for this symptom.

An allergic disease, such as atopic dermatitis, was considered rather unlikely, as the patient has not shown any signs of pruritus up to date.

In both human and veterinary medicine, PLE is a chronic, debilitating condition associated with recurrent skin infections [1,2,5,34]. In contrast to these experiences, our patient showed a mild course of disease that improved after a short course of oral prednisolone and remained in remission for more than a year after initial presentation, with the owner using only occasional lymphatic drainage massage as sole therapy for the condition.

To the authors‘ knowledge, this case report describes a novel presentation of a presumed congenital unilateral facial lymphedema of a young German Shorthair Pointer. This case demonstrates the value of using indirect CT lymphography in a case of localized lymphedema to identify the absence of enhancement of lymphatic vessels, as well as the lack of the drainage to regional lymph nodes for which a primary lymphedema is the most likely diagnosis.

## Data Availability

Not applicable.
